# Application of a Spacer-nick Gene-targeting Approach to Repair Disease-causing Mutations with Increased Safety

**DOI:** 10.21769/BioProtoc.4661

**Published:** 2023-04-20

**Authors:** Ngoc Tung Tran, Mikhail Lebedin, Eric Danner, Ralf Kühn, Klaus Rajewsky, Van Trung Chu

**Affiliations:** 1 Immune Regulation and Cancer, Max‐Delbrück‐Center for Molecular Medicine in the Helmholtz Association (MDC), Berlin, Germany; 2 Immune Mechanisms and Human Antibodies, Max‐Delbrück‐Center for Molecular Medicine in the Helmholtz Association (MDC), Berlin, Germany; 3 Genome Engineering & Disease Models, Max‐Delbrück‐Center for Molecular Medicine in the Helmholtz Association (MDC), Berlin, Germany

**Keywords:** Cas9^D10A^ nickase, PAM-out sgRNAs, Spacer distance of 200-350 bp, Gene repair approach, AAV6, Gene correction efficiency, Unintended on-target mutations, Off-target mutations, Human hematopoietic stem and progenitor cells (HSPCs)

## Abstract

The CRISPR/Cas9 system is a powerful tool for gene repair that holds great potential for gene therapy to cure monogenic diseases. Despite intensive improvement, the safety of this system remains a major clinical concern. In contrast to Cas9 nuclease, Cas9 nickases with a pair of short-distance (38–68 bp) PAM-out single-guide RNAs (sgRNAs) preserve gene repair efficiency while strongly reducing off-target effects. However, this approach still leads to efficient unwanted on-target mutations that may cause tumorigenesis or abnormal hematopoiesis. We establish a precise and safe *spacer-nick* gene repair approach that combines Cas9^D10A^ nickase with a pair of PAM-out sgRNAs at a distance of 200–350 bp. In combination with adeno-associated virus (AAV) serotype 6 donor templates, this approach leads to efficient gene repair with minimal unintended on- and off-target mutations in human hematopoietic stem and progenitor cells (HSPCs). Here, we provide detailed protocols to use the spacer-nick approach for gene repair and to assess the safety of this system in human HSPCs. The spacer-nick approach enables efficient gene correction for repair of disease-causing mutations with increased safety and suitability for gene therapy.

Graphical overview

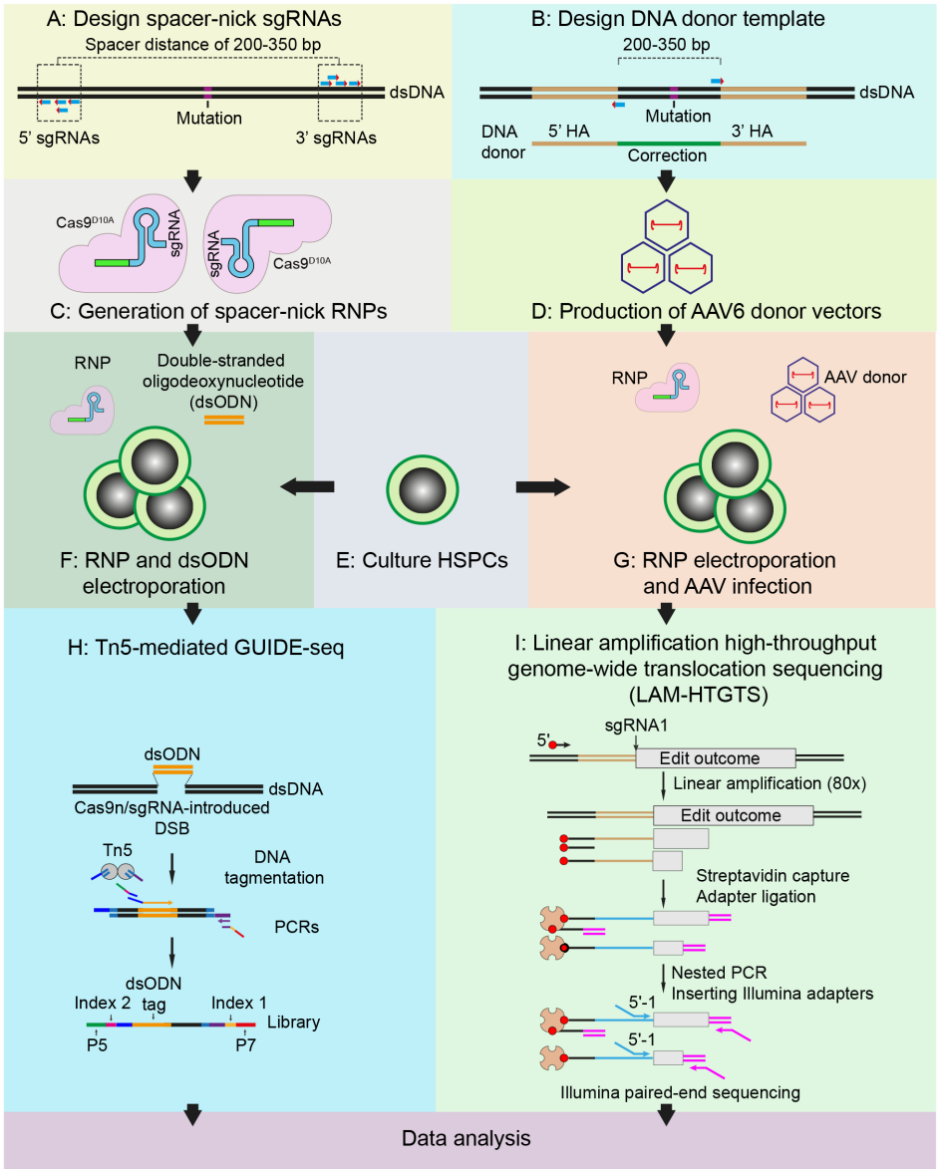

## Background

In the CRISPR/Cas9 system, a single-guide RNA (sgRNA)-directed Cas9 nuclease introduces double-strand breaks (DSBs) at the target region. DSBs are predominantly repaired by the non-homologous end joining (NHEJ) pathway, causing micro-insertions or deletions (indels or unwanted on-target mutations). If a DNA donor template with 5′ and 3′ homology arms (HAs) is provided, the homology-directed repair (HDR) pathway is activated to precisely replace the mutated DNA sequence (Cong et al., 2013; Hsu et al., 2013; Mali et al., 2013b; Chu et al., 2015). Although the CRISPR/Cas9 system succeeded in repairing mutations, its undesired on-target mutations and potential off-target activities are still a major concern (Cradick et al., 2013; Frock et al., 2015; Fu et al., 2013; Pattanayak et al., 2013; Tsai et al., 2015). The target specificity of CRISPR/Cas9 has been increased by using truncated sgRNAs or extended sgRNAs with two additional G nucleotides at the 5′ end (Cho et al., 2014; Fu et al., 2014; Tsai et al., 2015), sgRNAs with high specificity (Akcakaya et al., 2018), or high-fidelity SpCas9 mutants (Kleinstiver et al., 2016; Slaymaker et al., 2016; Vakulskas et al., 2018). A nickase, a mutated version of Cas9, can be generated by introducing a D10A or H840A mutation to RuvC (*E. coli* protein that is an endonuclease) or HNH (histidine-asparagine-histidine motif) nuclease domain, respectively (Jinek et al., 2012; Nishimasu et al., 2014). A sgRNA-guided Cas9 nickase introduces a nick at the target sequence, generating a single-stranded break that is perfectly repaired by the nonmutagenic base excision repair pathway (Dianov and Hubscher, 2013). As a result, Cas9 nickase creates less indels than the Cas9 nuclease. In combination with a pair of PAM-out (facing away from each other) sgRNAs at a short distance of 38–68 bp, the Cas9^D10A^ nickase generates offset double nicks that convert into site-specific DSBs. This approach led to efficient HDR- and NHEJ-mediated on-target events, while reducing off-target effects by 50–1,000-fold (Ran et al., 2013; Mali et al., 2013a). The limitation of this approach is that DSBs are still induced at the target sequence, leading to frequent indels and nonsense mutations in the target gene (Ran et al., 2013; Miyaoka et al., 2016).

Recently, we described a spacer-nick system that combines Cas9^D10A^ nickase with a pair of PAM-out sgRNAs at a long distance of 200–350 bp. In combination with adeno-associated virus (AAV) serotype 6 donor template delivery, the spacer-nick-based gene repair approach preserves efficient gene correction and minimizes adverse effects, such as unintended NHEJ-mediated on-target mutations and unwanted off-target genetic alterations in human hematopoietic stem and progenitor cells (HSPCs) (Tran et al., 2022). Additionally, we detailed modified GUIDE-seq and linear amplification high-throughput genome-wide translocation sequencing (LAM-HTGTS) methods to quantify genome-wide off-target mutations and capture all possible gene editing outcomes induced by sgRNA-guided nucleases in human HSPCs. The GUIDE-seq method is based on the integration of a blunt-end double-stranded (ds) oligodeoxynucleotide (dsODN) into the nuclease-induced DSBs and requires a large cell number (Tsai et al., 2015). To overcome this limitation and simplify this method to wet lab workflow, we modified it using Tn5 transposase to tagment genomic DNA and insert universal sequencing adapters into both ends of tagmented DNA fragments, allowing for an amplification of dsODN tags with specific primers (termed Tn5-mediated GUIDE-seq). Moreover, we modified the original HTGTS method previously described (Hu et al., 2016). This modification is based on linear amplification of primers outside of HAs and leads to a 5′- and 3′-based sequencing of the target region, allowing for a quantification of indels and deletion, AAV integrations, inversions, HDR, and translocations. Overall, we provide detailed step-by-step protocols for spacer-nick-mediated gene repair and off-target assessment in human HSPCs.

## Materials and Reagents

1. Pipette filter tips [Sarstedt, catalog numbers: 70.3010.275 (10 μL), 70.3030.265 (20 μL), 70.3030.375 (100 μL), 70.3030.110 (200 μL), 70.3060.275 (1,000 μL)]
2. 1.5 mL tubes (Eppendorf, catalog number: 0030 120.086)
3. 2.0 mL tubes (Eppendorf, catalog number: 0030 120.094)
4. 200 μL PCR plates (Sarstedt, catalog number: 72.1980.202)
5. 200 μL PCR tubes (Neolab, catalog number: 7-5207)
6. Agarose (Biozym, catalog number: 840004)
7. 100 bp DNA ladder (Thermo Fisher, catalog number: 5628019)
8. GeneRuler 1 kb Plus DNA ladder (Thermo Fisher, catalog number: SM1331)
9. AMPure XP beads (Beckman Coulter, catalog number: A63881)
10. Dynabeads^TM^ MyONE^TM^ Streptavidin C1 (Thermo Fisher, catalog number: 65001)
11. Nuclease-free water (Sigma, catalog number: 3098)
12. Ethanol (Carl Roth, catalog number: P075.1)
13. Tris (Carl Roth, catalog number: 5429.2)
14. HCl (Carl Roth, catalog number: 9277.2)
15. NaOH (Carl Roth, catalog number: P031.2)
16. EDTA·2H_2_O (Carl Roth, catalog number: 8043.3)
17. NaCl (Carl Roth, catalog number: 3957.4)
18. PEG 8000 (Sigma, catalog number: 89510-250G-F)
19. Human CD34^+^ microbead kit (Miltenyi Biotec, catalog number: 130-046-702)
20. Ficoll-Paque Plus (GE Healthcare, catalog number: 17144002)
21. Serum-free StemSpan^TM^ SFEM II medium (Stemcell, catalog number: 09655)
22. Human SCF (PeproTech, catalog number: 300-07)
23. Human TPO (PeproTech, catalog number: 300-18)
24. Human FLT3L (PeproTech, catalog number: 300-19)
25. Human IL-6 (PeproTech, catalog number: 200-06)
26. UM171 (Stemcell, catalog number: 72912)
27. StemRegenin 1 (SR1) (Stemcell, catalog number: 72342)
28. AAV6 system (Cell Biolabs, catalog number: VPK-410-SER6)
29. Fastdigest NotI (Thermo Fisher, catalog number: FD0593)
30. CrRNAs (IDT)
31. Alt-R^®^ CRISPR-Cas9 tracrRNA (IDT, catalog number: 1072534)
32. Alt-R^®^ S.p. Cas9^D10A^ nickase V3 (IDT, catalog number: 1081063)
33. P3 primary cell 4D-Nucleofector^TM^ X kit L (Lonza, catalog number: V4XP-3012)
34. Dead cell removal kit (Miltenyi Biotec, catalog number: 130-090-101)
35. KOD hot-start DNA polymerase (Merck Millipore, catalog number: 71085)
36. NucleoSpin Gel and PCR Cleanup (Macherey-Nagel, catalog number: 740609.50)
37. NucleoBond Xtra Maxi kit (Macherey-Nagel, catalog number: 740414.50)
38. GenFind V3 reagent kit (Beckman Coulter, catalog number: C34880)
39. Illumina Tagment DNA TDE1 Enzyme and Buffer kits (Illumina, catalog number: 20034197)
40. Zymo DNA clean and concentrator-5 (Zymo research, catalog number: D4003)
41. PrimeSTAR GXL polymerase (Takara, catalog number: R050B)
42. Qubit^TM^ dsDNA HS assay kit (Thermo Fisher, catalog number: Q32851)
43. High sensitivity D1000 ScreenTape assay (Agilent, catalog number: 5067)
44. QIAquick Gel Extraction kit (Qiagen, catalog number: 28704)
45. T4 DNA ligase (NEB, catalog number: M0202)
46. Hexaamminecobalt(III) chloride (HexCo) (Sigma, catalog number: 481521-25G)
47. Serum-free freezing medium BAMBANKER (Nippon Genetics, catalog number: 5802)
48. Phosphate-buffered saline (PBS) (Thermo Fisher, catalog number: 20012027)
49. Platinum Taq polymerase (Thermo Fisher, catalog number: 15966005)
50. Nextera XT index kit v2 set A (Illumina, catalog number: FC-131-2001)
51. 5 M Tetramethylammonium chloride solution (TMAC) (Sigma, catalog number: T3411-500ML)
52. Gel Loading Dye, Orange (6×) (NEB, catalog number: B7022S)
53. Q5 High-fidelity DNA polymerase (NEB, catalog number: M0491S)
54. Wizard Genomic DNA purification kit (Promega, catalog number: A1120)
55. ProNex size-selective purification beads (Promega, catalog number: NG2001)
56. High sensitivity DNA BioAnalyzer kit (Agilent, catalog number: 5067-4626)
57. TOP10 bacteria (Invitrogen, catalog number: C404003)
58. LB agar, powder (Invitrogen, catalog number: 22700025)
59. Carbenicillin (Sigma, catalog number: C1613-1ML)
60. T4 DNA ligase (NEB, catalog number: M0202M)
61. P3 Primary Cell electroporation buffer (see Recipes)
62. Completed serum-free StemSpan^TM^ SFEM II medium (see Recipes)
63. 70% ethanol (see Recipes)
64. 50% (wt/vol) PEG 8000 (see Recipes)
65. 5 M NaCl (see Recipes)
66. 2.5 N NaOH (see Recipes)
67. 0.5 M EDTA (see Recipes)
68. 1 M Tris-HCl (pH 7.4) (see Recipes)
69. 1 M Tris-HCl (pH 8.0) (see Recipes)
70. 1× TE buffer (pH 8.0) (see Recipes)
71. B&W buffer (see Recipes)
72. 20 mM hexaamminecobalt(III) chloride (see Recipes)
73. 50 mM bridge adapter (see Recipes)
74. Forward and reverse oligos of dsODN (IDT) (Table 1)
75. Primers (Eurofins) (Table 1)
  **Table 1.** Oligos and primers

Name	Sequence
dsODN-forward	5′-P-G*T*TTAATTGAGTTGTCATATGTTAATAACGGT*A*T-3′
dsODN-reverse	5′-P-A*T*ACCGTTATTAACATATGACAACTCAATTAA*A*C-3′
I5-Nextera-3′-GSP (-)	TCGTCGGCAGCGTCAGATGTGTATAAGAGACAGGTTTAATTGAGTTGTCATATGTTAATAACGGT
I5-Nextera-5′-GSP (+)	TCGTCGGCAGCGTCAGATGTGTATAAGAGACAGATACCGTTATTAACATATGACAACTCAATTAA
I7-Nextera-reverse	GTCTCGTGGGCTCGGAGATGTGTATAAG
Bridge-lower	GCGACTATAGGGCACGCGTGGNNNNNN[AmC3]
Bridge-upper	[Phos]CCACGCGTGCCCTATAGTCGC[AmC3]
U2-C-Adapter	GTCTCGTGGGCTCGGAGATGTGTATAAGAGACAGNNNNNGACTATAGGGCACGCGTGG
U1-C-GeneSpecific	TCGTCGGCAGCGTCAGATGTGTATAAGAGACAGNNNNN[GeneSpecific]
FC1-i5-U1 (N501)	AATGATACGGCGACCACCGAGATCTACAC tagatcgc TCGTCGGCAGCGTC
FC2-i7-U2 (N701)	CAAGCAGAAGACGGCATACGAGAT tcgcctta GTCTCGTGGGCTCGG

## Equipment

1. 96-well magnet stand (Thermo Fisher, catalog number: AM10027)
2. 6-well magnet stand (Cytiva, catalog number: 28948964)
3. Tissue culture 6-well plates (Sarstedt, catalog number: 83.3920)
4. Tissue culture 12-well plates (Sarstedt, catalog number: 83.3921)
5. Freezing vials (Sarstedt, catalog number: 72.380.992)
6. 16-well nucleocuvette strip (Lonza, catalog number: V4XP-3032)
7. Lonza 4D-Nucleofector (Lonza, catalog number: AAF-1003B)
8. Pipettes (Gilson)
9. Multichannel pipettes (Eppendorf)
10. PCR thermocycler (Eppendorf, catalog number: 6331000017)
11. NanoDrop (Thermo Fisher)
12. Thermomixer (Eppendorf)
13. Vortexer (Scientific Industries)
14. Centrifuge (Eppendorf)
15. Tissue culture incubator set at 37 °C, 5% CO_2_ (Binder)
16. TapeStation (Agilent, catalog number: G2991BA)
17. Qubit 2.0 fluorometer (Invitrogen, catalog number: Q32866)
18. ChemiDoc XRS gel imaging system (Bio-Rad)
19. Standard UV transilluminator (Cleaver Scientific)
20. TC20 automated cell counter (Bio-Rad, catalog number: 1450102)
21. Tube roller (Starlab, catalog number: N2400-7010)
22. 2100 BioAnalyzer instrument (Agilent, catalog number: G2939BA)

## Software

1. CrispRGold (the MDC, https://crisprgold.mdc-berlin.de)
2. R 4.0.3 programming language (R-project, https://www.r-project.org/)
3. R-studio integrated development environment (R-studio, https://www.rstudio.com/)
4. GelAnalyzer 19.1 gel densitometry software (GelAnalyzer, http://www.gelanalyzer.com/?i=1)

## Procedure

- **Design spacer-nick sgRNAs**
*Note: For testing gene editing efficiencies of sgRNAs, you can select any available system that is suitable for your laboratory, such as T7EI (T7 endonuclease I) or Sanger sequencing following ICE analysis. Additionally, sgRNAs could be ordered as a pair of oligos, cloned into expression vectors, and tested in human cell lines such as HEK293T. You can also order synthetic sgRNAs, generate pre-assembled ribonucleoprotein (RNP) complexes of Cas9 nuclease and synthetic sgRNA, and perform in vitro T7EI assay on PCR amplicons.*
  1. Define 5′ and 3′ areas flanking mutation hotspots (Figure 1) with a spacer distance of 200–350 bp.
    **Figure 1.**
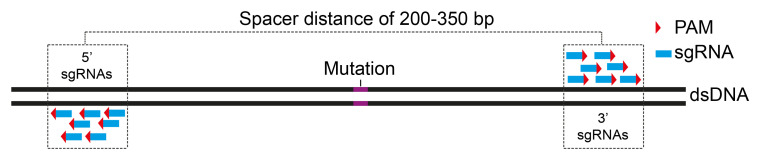
Design pools of spacer-nick sgRNAs. Scheme showing selected areas with a spacer distance of 200–350 bp, flanking a mutation. Pools of 5′ and 3′ sgRNAs with configurations of PAM sites facing outwards (PAM-out) are designed.
  2. Design pools of sgRNAs on the selected areas with a PAM-out configuration using CrispRGold software. In combination with the Cas9^D10A^ nickase, 5′ sgRNAs nick the plus strand, whereas 3′ sgRNAs nick the opposite strand of the dsDNA molecule.
  3. Order designed sgRNAs, clone them into the suitable plasmids, and test gene editing efficiencies of these sgRNAs using T7EI assay or Sanger sequencing following ICE analysis.
  4. Select several pairs of PAM-out sgRNAs (one 5′ sgRNA and one 3′ sgRNA) with equal and high gene editing efficiencies (>80%) for the spacer-nick gene repair approach.
- **Design DNA donor templates**
*Note: In order to quantify gene correction efficiencies, we insert a diagnostic restriction enzyme site by introducing silent mutations into correct sequences or develop correct integration PCR, which allows us to amplify repaired and non-repaired alleles. To clone DNA donor fragments into the AAV genome vector, we inserted NotI sites into both ends of DNA donor fragments together with six random nucleotides.*
  1. Design a DNA donor template carrying 5′ and 3′ HAs of at least 0.5–1.5 kb outside of each nick site (Figure 2).
  2. In order to minimize a mini-homologous sequence (highlighted in green; Figure 2) between two nick sites, we modify this sequence by introducing silent mutations (in case of repairing mutations in coding exons) or by partially ablating this sequence (in case of inserting cDNA into loci).
    **Figure 2.**
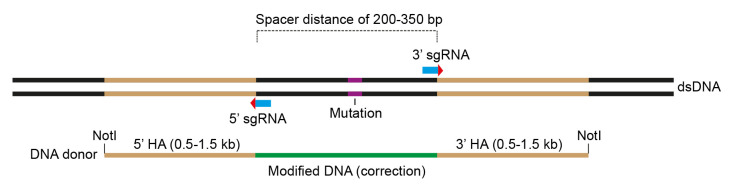
Design the DNA donor template. Scheme showing configuration of the DNA donor template that carries 5′ and 3′ homology arms, flanking a modified sequence (marked in green) for gene correction and NotI sites at both ends for cloning into the pAAV vectors.
  3. Add NotI restriction enzyme sites to both ends of the DNA donor template.
  4. Order the dsDNA template from IDT.
- **Generation of spacer-nick RNPs**
*Note: We calculated the amounts of sgRNAs and Cas9^D10A^ nickase for a reaction of 20 μL electroporation. For a reaction of 100 μL electroporation, scale up proportionally.*
  1. Order crRNAs, tracrRNA, and Cas9^D10A^ nickase from IDT.
  2. Generate sgRNA complex by mixing 20 μL of each crRNA (100 pmol) with 20 μL of tracrRNA (100 pmol) at a molarity ratio of 1:1 in a PCR tube.
  3. Incubate the mixture at 95 °C for 5 min and ramp down at a rate of 1 °C/s to room temperature in the thermocycler.
  4. Store sgRNA complexes at -20 °C up to six months or -80 °C up to one year.
  5. Generate RNP complexes by mixing 2 μL of sgRNA (100 pmol) with 0.75 μL of Cas9^D10A^ nickase (~50 pmol) at a molarity ratio of 2:1 in a PCR tube and incubate the mixture at 25 °C for 10 min in the thermocycler.
*Note: Assembled RNPs should be freshly made before use.*
- **Production and purification of AAV6 donor vectors**
  *Note: We recommend using high-fidelity KOD hot-start DNA polymerase for PCR amplification of the DNA donor template. However, you can use any high-fidelity DNA polymerases that are available in your laboratory. Production and purification of AAV6 donor vectors are described in detail in our previous protocol (Tran et al., 2020)*.
  1. Amplify the NotI-containing dsDNA donor template (step B4) with specific forward and reverse primers (carrying six random nucleotides, NotI site, and target-specific sequence) using any high-fidelity DNA polymerases.
  2. Purify PCR products using NucleoSpin Gel and PCR Cleanup kit.
  3. Digest PCR products with NotI restriction enzyme and load on 0.8% agarose gel.
  4. Cut digested dsDNA band and purify it using any gel extraction kit.
  5. Clone the purified dsDNA template into NotI-linearized pAAV vectors using T4 DNA ligase.
  6. Transform the cloned pAAV vectors into competent TOP10 bacteria using heat-shock method.
  7. Spread transformed bacteria on 10 cm LB agar dishes containing 50 μg/mL of carbenicillin and culture these dishes in a bacterial incubator at 30 °C overnight.
  8. Pick bacterial colonies and inoculate these with 2 mL of liquid LB medium containing 50 μg/mL of carbenicillin at 30 °C overnight in a bacterial incubator with shaker.
  9. Extract plasmids using any plasmid purification kit and confirm the insert by Sanger sequencing.
  10. Produce large quantities of pAAV donor vectors using NucleoBond Xtra Maxi kit.
  11. Transfect, purify, concentrate, and calculate copy number of AAV6 donor particles following our previous protocol (Tran et al., 2020).
- **Culture human HSPCs**
*Note: In order to achieve sufficient expansion of human CD34^+^ cells, we recommend that you culture ~4 × 10^5^ HSPCs per 2 mL in a well of a 6-well plate and monitor the culture every day until these cells are used for electroporation. In case expanded cells are at high density, you should split them into new wells of a 6-well plate.*
  1. Isolate human CD34^+^ HSPCs from mobilized peripheral blood or bone marrow of healthy donors or patients using Ficoll-Paque Plus and follow human CD34+ microbead kit according to the manufacturer’s protocol.
  2. Freeze isolated CD34^+^ HSPCs in serum-free freezing medium (BAMBANKER) at a density of 1 × 10^6^ per milliliter and store in liquid nitrogen for long-term storage.
  3. Thaw frozen vials and culture CD34^+^ HSPCs at a density of 4 × 10^5^ per 2 mL of completed serum-free StemSpan^TM^ SFEM II medium in a well of a 6-well plate.
  4. Exchange half of the old medium with new medium 24 h post culture.
  5. Seventy-two hours post culture, harvest expanded HSPCs and count cell number using TC20 cell counting system.
  6. Transfer 2 × 10^5 ^HSPCs to a 1.5 mL Eppendorf tube and spin down at 300 × *g* for 5 min.
  7. Remove supernatant, wash the cell pellet two times with room temperature PBS, and proceed to Section F or Section G.
- **RNP and dsODN electroporation for Tn5-mediated GUIDE-seq**
*Note: Prepare 1 mL of pre-warmed medium by adding 1 mL of completed serum-free StemSpan^TM^ SFEM II medium to a well of a 12-well plate and place it into an incubator at 37 °C prior to electroporation. For long-term storage, annealed dsODN should be stored at -80 °C.*
  1. Order forward and reverse oligos of dsODN as previously described (Tsai et al., 2015) from IDT.
  2. Anneal two oligos of dsODN by mixing 50 μL of each oligo (100 pmol), incubate at 95 °C for 5 min, and ramp down at a rate of 1 °C/s to room temperature in the thermocycler.
  3. After the last wash with PBS in step E7, remove supernatant and resuspend the cell pellet with 20 μL of P3 Primary Cell electroporation buffer by pipetting up and down 10 times.
  4. Add 2.75 μL of 5′ and 3′ assembled RNPs (step C5) and 0.5 μL (25 pmol) of the annealed dsODN to cell suspension and mix well by pipetting up and down five times.
  5. Transfer the mixture to a well of a 16-well nucleocuvette strip.
  6. Electroporate the cells using the DZ-100 program of Lonza 4D-Nucleofector.
  7. Transfer the electroporated cells to pre-warmed serum-free StemSpan^TM^ SFEM II medium.
  8. Place the cell plate into an incubator at 37 °C and 5% CO_2_.
  9. Change medium every 2–3 days.
  10. At day 10 post electroporation, harvest the edited HSPCs by pipetting up and down five times, transfer cell suspension to a 15 mL Falcon tube, and spin down at 300 × *g* for 5 min.
  11. Wash the cell pellet three times with room temperature PBS.
  12. Deplete the dead cells by using dead cell removal kit and follow manufacturer’s specifications.
  13. Isolate gDNA using GenFind V3 reagent kit according to the manufacturer’s protocol.
  14. Measure concentration of gDNA using NanoDrop and dilute gDNA to 20 ng/μL.
  15. Check quality of gDNA by loading 500 ng of gDNA on 0.8% agarose gel (Figure 3) and proceed to Section H.
*Note: In order to achieve good results of Tn5-mediated DNA tagmentation, we recommend using GenFind V3 kit for gDNA isolation. This kit yields high quality of gDNA, and DNA fragment size is >20 kb (Figure 3). However, you can also use other gDNA isolation kits with equal quality that are available in your laboratory.*
    **Figure 3.**
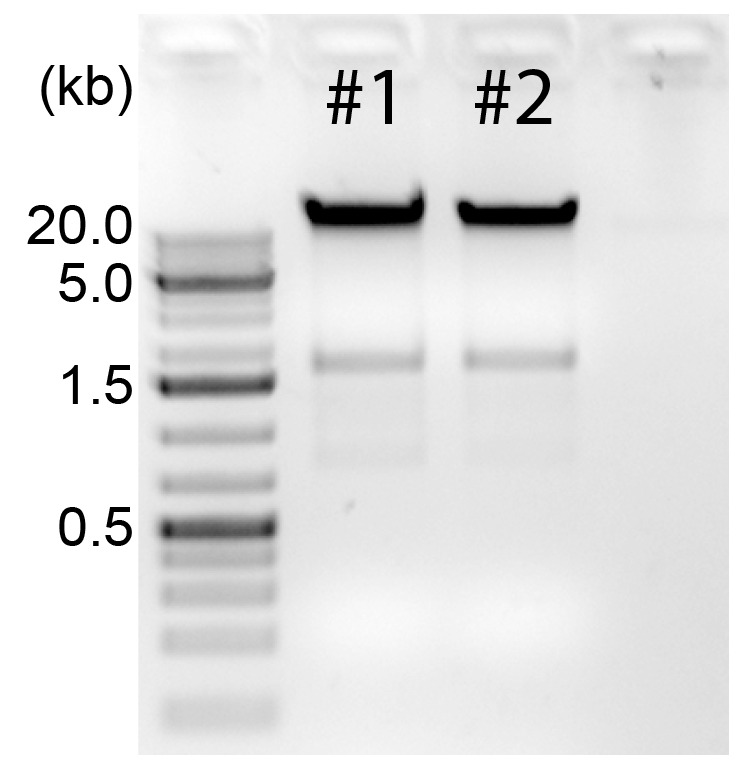
Quality control of the genomic DNA. 500 ng of gDNA of samples #1 and #2 are loaded on 0.8% agarose gel. GeneRuler 1 kb Plus DNA ladder is used as a marker. Optimal length of gDNA fragments is >20 kb.
- **RNP electroporation and AAV6 infection for LAM-HTGTS**
*Note: We normally infect the electroporated HSPCs with rAAV6 donor vectors at a MOI of 1 × 10^5^ genome copy/cell. Higher MOI leads to cell death; we recommend carefully titrating your rAAV donor particles.*
  1. At the last wash with PBS in step E7, remove supernatant and resuspend 2 × 10^5 ^HSPCs with 20 μL of P3 Primary Cell electroporation buffer by pipetting up and down 10 times.
  2. Add 2.75 μL of 5′ and 3′ spacer-nick RNPs (step C5) to cell suspension and mix well by pipetting up and down five times.
  3. Transfer the mixture to a well of a 16-well nucleocuvette strip.
  4. Electroporate the cells using the DZ-100 program of Lonza 4D-Nucleofector.
  5. Transfer the electroporated cells to pre-warmed completed serum-free StemSpan^TM^ SFEM II medium.
  6. Place the plate into an incubator at 37 °C and 5% CO_2_.
  7. Fifteen to thirty minutes later, add rAAV6 donor particles to the well containing the electroporated cells at a MOI of 1 × 10^5^ genome copy/cell.
  8. Change half the medium with new completed serum-free StemSpan^TM^ SFEM II medium 6–8 h after AAV infection.
  9. Change medium every 2–3 days.
  10. At day 18 post infection, harvest the cells by pipetting up and down five times, transfer cell suspension to a 15 mL Falcon tube, and spin down at 300 × *g* for 5 min.
  11. Wash the cell pellets three times with room temperature PBS.
  12. Deplete dead cells by using dead cell removal kit following manufacturer’s specifications.
  13. Isolate gDNA using Wizard Genomic DNA purification kit (see the note at step F15) and proceed to Section I.
- **Tn5 transposase-mediated GUIDE-seq**
*Note: Accurate concentration of gDNA is essential for reproducible Tn5-mediated tagmentation. We describe a Tn5-tagmented DNA protocol for exactly 100 ng of gDNA. If you want to conduct the protocol for more or less than 100 ng of gDNA, you should optimize these conditions. The Tn5-mediated DNA tagmentation should produce DNA fragments in the range of 300–1,500 bp. If DNA fragments are smaller or larger than this, you should reduce or increase the incubation time for the tagmentation, respectively. Importantly, you must carry out the Tn5-mediated DNA tagmentation at room temperature; do not use vortex for mixing. Primers used in this protocol are the same as described in Tran et al. (2022).*
  1. Add the following reagents stepwise to a well of an 8-well PCR strip as in Table 2:
    **Table 2.** Tn5-mediated DNA tagmentation

Steps	Reagents	Volume (µL)
1	Nuclease-free water	3
2	gDNA (20 ng/µL) (Section F)	5
3	2× TD buffer	10
4	TDE1 (Tn5) enzyme	2
Total		20
  2. Mix carefully by pipetting up and down 10 times, avoiding bubbles.
  3. Close the PCR strip and centrifuge briefly at 280 × *g* for 30 s.
  4. Incubate the PCR strip on the thermocycler at 55 °C for 7 min.
  5. Transfer the PCR strip on ice.
  6. Clean up Tn5-tagmented DNA fragments using Zymo DNA clean and concentrator-5 according to the manufacturer’s protocol.
  7. Elute tagmented DNA fragments with 15 µL of nuclease-free water.
  8. Check quality of tagmented DNA fragments by loading 3 µL of the elute on 1.2% agarose gel (see Figure 4) and proceed to step H9.
    **Figure 4.**
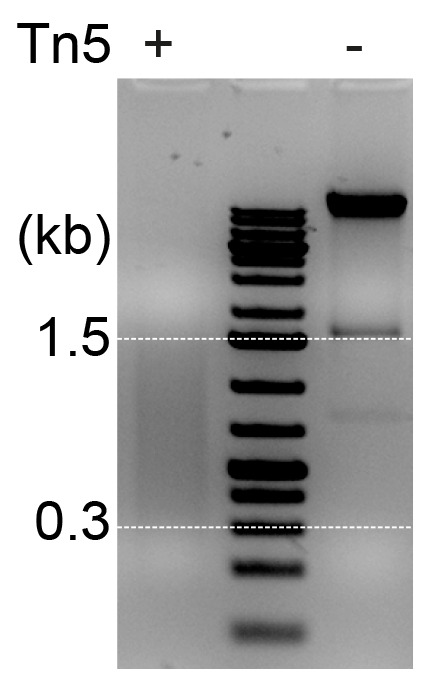
Verification of tagmented DNA fragments. Agarose gel analysis showing fragmentation of gDNA treated with (+) or without (-) Tn5 transposase. The Tn5-mediated tagmentation should produce DNA fragments in the range of ~0.3–1.5 kb (marked by white dash lines).
  9. Prepare first PCR in a well of a 96-well plate according to Table 3.
    **Table 2.** Amplifying dsODN-tagged amplicons

Reagents	Volume (µL)
10× buffer (MgCl_2_ free)	3
MgCl_2_, 50 mM	1.2
dNTP mix, 10 mM	0.6
Platinum Taq polymerase (5 U/µL)	0.3
TMAC (0.5 M)	1.5
I5-Nextera-3′-GSP (-) or 5′-GSP (+)-forward (10 μM)	1.5
I7-Nextera-reverse (10 μM)	0.75
Tagmented DNA (step H7)	10
Nuclease-free water	11.15
Total	30
  10. Spin the plate at 280 × *g* for 1 min.
    - Run the plate on the thermocycler with the touchdown program at the following conditions:
    - 95 °C for 5 min.
    - 15 cycles of [95 °C for 30 s, 70 °C (-1 °C/cycle) for 2 min, 72 °C for 30 s].
    - 10 cycles of (95 °C for 30 s, 55 °C for 1 min, 72 °C for 30 s).
    - 72 °C for 5 min.
    - 4 °C hold.
  11. Purify and clean up PCR products using AMPure XP beads (1.2×; 1.2 μL beads per 1 μL of sample) as follows:
    - Add 36 μL of AMPure XP beads to a PCR reaction.
    - Mix well by pipetting up and down 15 times using a 200 μL multichannel pipette.
    - Incubate the PCR plate at room temperature for 5 min.
    - Place the PCR plate on a 96-well magnetic stand for 5 min or until the solution is clear.
    - Remove the supernatant using the multichannel pipette.
    - Take the PCR plate out of the magnetic stand, add 200 μL of freshly made 70% ethanol to the beads, and mix by pipetting up and down.
    - Put the plate on the magnetic stand for 2 min and remove the supernatants.
    - Repeat steps f–g one more time.
    - Let the beads air dry for 3–5 min.
    - Take the plate out of the magnetic stand, add 18 μL of nuclease-free water, mix the beads, and incubate at room temperature for 2 min.
    - Put the plate on the magnetic stand for 5 min.
    - Transfer 15 μL of eluted DNA to a new well of a 96-well PCR plate and proceed to step H12.
  12. Prepare second index PCR in a well of a 96-well plate according to Table 4.
    **Table 4.** Indexing PCR fragments

Reagents	Volume (µL)
Nuclease-free water	2.4
10× buffer for Platinum Taq Mg^2+ ^free	3
50 mM Mg^2+^	1.2
Platinum Taq (5 U/μL)	0.3
dNTP 10 mM	0.6
TMAC (0.5 M)	1.5
Nextera-I5 barcode-primer (10 μM)	3
Nextera-I7 barcode-primer (10 μM)	3
1^st^ PCR product	15
Total	30
  13. Run the plate on the thermocycler with touchdown program at the following conditions:
    - 95 °C for 5 min.
    - 15 cycles of [95 °C for 30 s, 70 °C (-1 °C/cycle) for 2 min, 72 °C for 30 s].
    - 10 cycles of (95 °C for 30 s, 55 °C for 1 min, 72 °C for 30 s).
    - 72 °C for 5 min.
    - 4 °C hold.
  14. Purify and clean up PCR products using AMPure XP beads (0.7×, see above).
  15. Elute PCR products with 30 µL of 1× TE buffer (pH 8.0).
  16. Verify size of PCR products by loading 3 µL of the eluate on a 1.5% agarose gel (Figure 5).
    **Figure 5.**
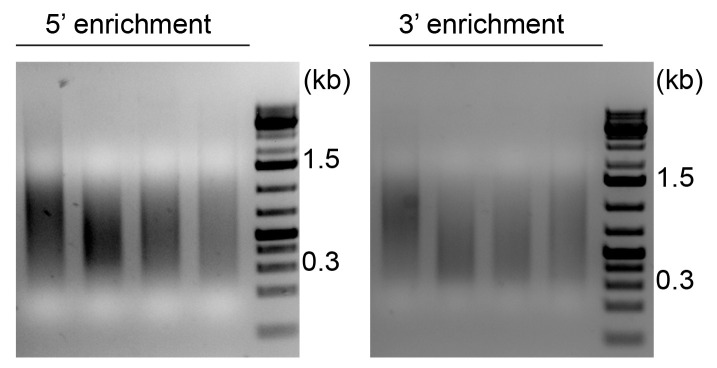
Verification of 5′ and 3′ enriched PCR products. Agarose gel analysis showing 5′ (left) and 3′ (right) enriched PCR products. Size range of PCR products is approximately 0.3–1.5 kb.
  17. Measure DNA concentration using Qubit dsDNA HS high sensitivity kit following manufacturer’s specifications.
  18. Calculate molar amount of each DNA library based on the size and concentration.
  19. Pool equimolar amount of each DNA library into a 2 mL Eppendorf tube.
  20. Clean and concentrate the pooled DNA library using AMPure XP beads (0.9×, see above).
  21. Elute the pooled DNA library with 40 μL of nuclease-free water.
  22. Save 2 μL of the elute for final quality control.
  23. Mix 35 μL of the pooled DNA library with 7 μL of Gel Loading Dye, Orange (6×).
  24. Load the mixture on two lanes (21 μL per lane) of 1.2% agarose gel.
  25. Load 4 μL of 100 bp DNA ladder onto left and right lanes flanking the sample lanes.
  26. Run the gel at 120 V for 30 min.
  27. Under a standard UV transilluminator, cut out bands in a range of 350–1,000 bp (Figure 6).
    **Figure 6.**
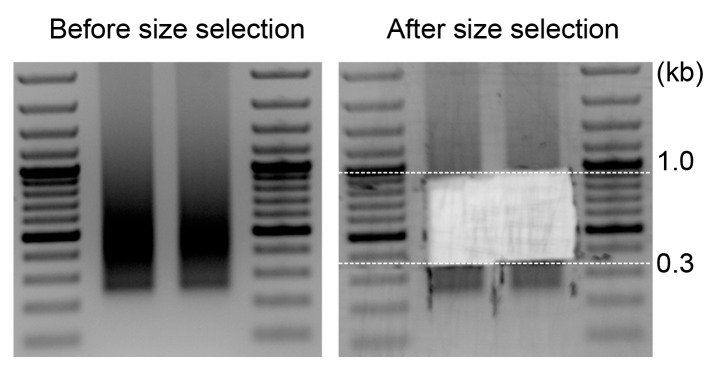
Before and after size selection of the final library. Agarose gel images showing the DNA library before and after size selection. Size range (0.35–1.0 kb) of DNA fragments is excised and the DNA is extracted from the gel.
  28. Extract DNA using QIAquick Gel Extraction kit following the manufacturer’s specifications.
  29. Elute DNA with 100 μL of nuclease-free water.
  30. Pool two lanes into a 2 mL Eppendorf tube.
  31. Clean up and concentrate the final DNA library with AMPure XP beads (0.9×).
  32. Elute DNA with 30 μL of 1× TE buffer (pH 8.0).
  33. Measure DNA concentration of the final DNA library using Qubit dsDNA high sensitivity kit following manufacturer’s specifications.
  34. Verify size range of the eluted DNA (step H22) and the final DNA library (step H32) using the D1000 ScreenTape kit (Figure 7).
    **Figure 7.**
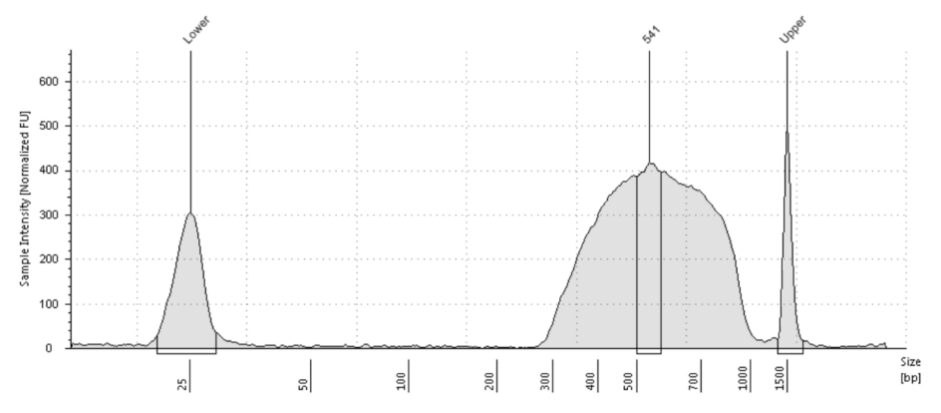
TapeStation analysis for the final DNA library. The TapeStation analysis verifying length of the final DNA library. Size range of the DNA library is approximately 300–1,000 bp with a peak of 541 bp.
  35. Calculate molar amount of the final DNA library.
  36. Dilute the final DNA library to 4 nM with 1× TE buffer (pH 8.0).
  37. Proceed to Illumina deep sequencing.
- **LAM-HTGTS**
*Note: Contamination of low molecular weight DNA fragments (<1 kb) will inhibit the LAM-HTGTS; therefore, it is crucial to remove any <1 kb fragments by cleaning gDNA up with ProNex size-selective purification beads. It is imperative to optimize the linear amplification PCR for it not to amplify any double-stranded products that will block the generation of the linear amplicons and the adapter ligation.*
  1. Ensure the high quality of gDNA by loading 50–100 ng on 0.8% agarose gel. If any <1 kb fragments are detected (Figure 8), purify the gDNA with 1.0× ProNex size-selective purification beads.
    **Figure 8.**
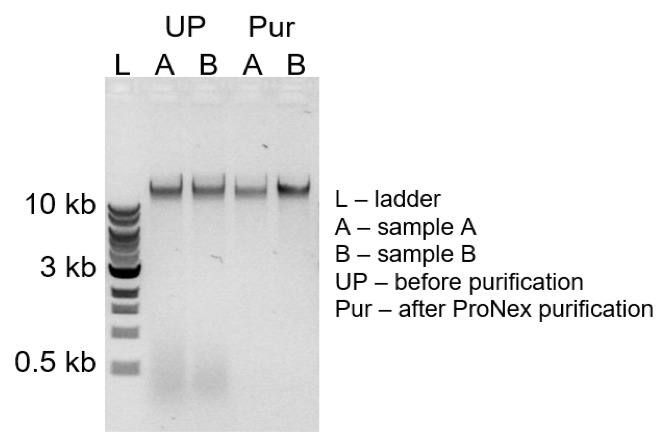
Quality control of the genomic DNA. Agarose gel image showing quality of gDNA before (UP) and after (PUR) ProNex bead purification. gDNA (UP: A and B) contains <1 kb fragments that are successfully removed by 1.0× ProNex beads purification (Pur: A and B).
  2. Prepare linear amplification PCR using PrimeSTAR GXL DNA polymerase as in Table 5.
    **Table 5.** Linear amplification PCR

Reagents	Volume (µL)
Nuclease-free water	28.5
5× GXL buffer	10
dNTPs 2.5 mM each	4
Biotin-conjugated gene specific primer (1 µM)	2
Genomic DNA (30 ng/µL) (step G13)	5
GXL polymerase	0.5
Total	50
  3. Mix the reaction by vortexing and spin down at 280 × *g* for 1 min.
  4. Perform the amplification on the thermocycler using the following program:
    - 98 °C for 5 min.
    - 80 cycles of (98 °C for 30 s, 60 °C for 30 s, 68 °C 90 s).
    - 68 °C for 2 min.
    - 4 °C hold.
*Note: Important for the first time, optional for the established reactions: check 5 μL of the reaction on 0.8% agarose gel to ensure there was no exponential off-target amplification.*
  5. Proceed with biotinylated PCR product capture through the following steps:
    - Transfer 2 μL of Dynabeads^TM^ MyONE^TM^ Streptavidin C1 per each PCR reaction into a 1.5 mL Eppendorf tube and add 150 μL of B&W buffer to the tube.
    - Put the tube on the magnetic stand (6-well magnetic rack) for 5 min.
    - Remove the supernatant and add 150 μL of B&W buffer to the tube.
    - Take the tube out of the magnetic stand and resuspend the beads by pipetting up and down.
    - Put the beads on the magnetic stand for 5 min and remove the supernatant.
    - Resuspend the beads in 2 μL of nuclease-free water per each PCR reaction and mix the reactions as shown in Table 6.
      **Table 5.** Capturing biotinylated PCR products

Reagents	Volume (µL)
LAM-PCR product (step I4)	50
5 M NaCl	14
0.5 M EDTA	0.7
Washed StrepBeads	2
Nuclease-free water	3.3
Total	70
    - Incubate the reaction on the tube roller for 2–4 h.
*Note: Four hours is recommended, but the reaction can be rolled overnight.*
    - Capture the DNA–bead complexes on the magnetic stand.
    - Remove the supernatant and wash the beads with 150 μL of B&W buffer three times.
    - Wash the beads with 150 μL of nuclease-free water.
    - Resuspend the beads in 9 μL of nuclease-free water.
  6. Proceed with on-bead ligation steps as follows:
    - Prepare and mix the reagents as in Table 7.
      **Table 7.** On-bead ligation

Reagents	Volume (µL)
DNA–Bead complexes (step I5k)	4.5
10× T4 DNA ligase buffer (NEB)	1
BridgeAdapter 50 mM (see Recipes)	0.5
T4 DNA ligase (NEB)	0.5
HexCo 20 mM	0.5
50% PEG8000 (see Recipes)	3
Total	10

*Note: Add the DNA–bead complexes first to the reaction, before adding 50% PEG8000. Mix the reaction thoroughly by pipetting and vortexing.*
    - Incubate the reactions in the thermocycler with the following program: 22 °C for 1 h, 16 °C for 1 h, 14 °C for 1 h, 10 °C for 1 h, and store at 4 °C.
    - Capture the DNA–bead complexes on the magnetic stand.
    - Remove the supernatant and wash the beads with 150 μL of B&W buffer three times.
    - Wash the beads with 150 μL of nuclease-free water.
    - Resuspend the beads in 20 μL of nuclease-free water.
  7. Prepare and mix reagents for on-bead adapter PCR as in Table 8:
    **Table 8.** Adapter PCR

Reagents	Per reaction (µL)
Nuclease-free water	13.75
5× Q5 buffer	5
dNTPs	2
U1-C-Gene specific nested forward (10 μM)	1
U2-C-AdPrim (10 μM)	1
DNA–Bead complexes	2
Q5 polymerase	0.25
Total	25
  8. Perform on-bead adapter PCR at the following conditions:
    - 98 °C for 5 min.
    - 30 cycles of (98 °C for 30 s, 60–65 °C 30 s, 72 °C 90 s).
    - 72 °C for 2 min.
    - 4 °C hold.
  9. Load 5 μL of the reaction on 2% agarose gel along with the 100 bp ladder. The peak of the length distribution should be in the range of 300–500 bp (Figure 9).
    **Figure 9.**
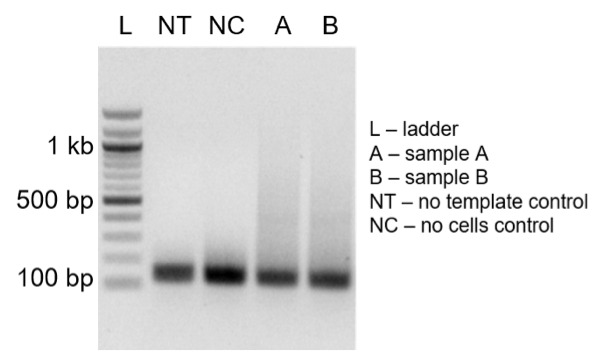
Verification of on-bead adapter PCR products. Agarose gel analysis showing size range of on-bead adapter PCR products. The length distribution of on-bead adapter PCR products (A and B) should be in the range of 300–500 bp.
  10. Purify PCR products with ProNex beads (1.2×) and proceed to Illumina library preparation.
  11. Tag different samples with Illumina barcode sequences by mixing the reagents as in Table 9:
    **Table 9.** Indexing PCR

Reagents	Per reaction (µL)
Nuclease-free water	10.75
5× Q5 buffer	5
dNTPs	2
FC1-i5-U1 (10 μM)	1
FC2-i7-U2 (10 μM)	1
Purified PCR product from step I10	5
Q5 polymerase	0.25
Total	25
  12. Perform indexing PCR at the following conditions:
    - 98 °C for 5 min.
    - 5–10 cycles of (98 °C for 30 s, 55 °C 30 s, 72 °C 90 s).
    - 72 °C for 2 min.
    - 4 °C hold.
  13. Load 5 μL of the reaction on 2% agarose gel along with the 100 bp ladder. The peak of the length distribution should be around 300–500 bp (Figure 10).
    **Figure 10.**
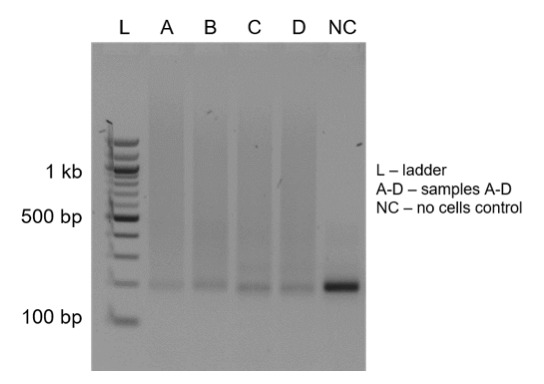
Verification of indexed PCR products. Agarose gel analysis showing the length distribution of indexed PCR amplicons (samples: A to D) with a smear peaking at 200–1,000 bp. A negative control (NC) is used as a loading control.
  14. Quantify the concentration of the products 500–700 bp long using densitometry in GelAnalyzer software.
  15. Pool the samples so that the mass of the product per mass of gDNA used as an input (or number of the cells) is equal, to ensure a homogenous variant coverage.
  16. Load 50–100 μL of the reaction on 0.8% agarose gel along with the 100 bp ladder and cut the 500–1,000 bp smear from the gel.
  17. Extract the product from the agarose using QIAquick gel extraction kit. Measure the concentration on Nanodrop.
  18. Ensure the proper size selection by loading the library diluted to 10 ng/μL on High Sensitivity D1000 ScreenTape or High Sensitivity dsDNA chip of BioAnalyzer (Figure 11).
    **Figure 11.**
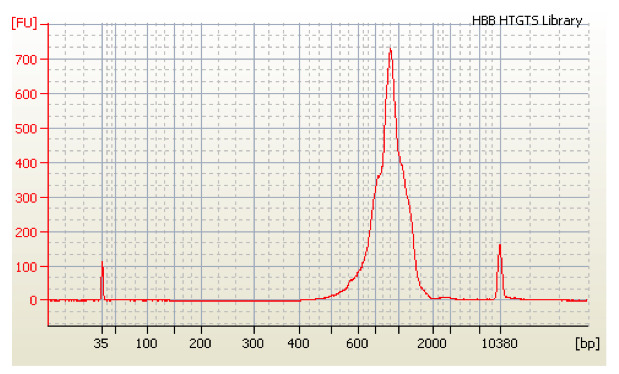
BioAnalyzer track for the final library. The size distribution and molarity of the final DNA library are verified by the high sensitivity BioAnalyzer dsDNA chip. The length of the DNA library is in the range of 600–1,200 bp.
  19. Calculate the molarity of the sample using ScreenTape or BioAnalyzer software and proceed with the library loading as described in the corresponding Illumina kit manufacturer’s manual.

## Data analysis

For GUIDE-seq experiments, three independent experiments with two replicates each were performed. For LAM-HTGTS experiments, 3–6 independent experiments with two replicates each were performed. The pipelines for analyzing the GUIDE-seq and LAM-HTGTS were based on previous publications (Giannoukos et al., 2018; Danner et al., 2021). Detailed analysis of GUIDE-seq and LAM-HTGTS has been described in the Methods section and the supplementary figure S9 of the original paper (Tran et al., 2022). The Jupiter notebooks, conda environment, and scripts are available on https://github.com/ericdanner/SpacerNick. Briefly, GUIDE-seq reads were checked for correct priming and the sequence of the dsODN was trimmed to adjacent genomic sequences that are globally mapped to human genome (hg38) using Bowtie2. Mapped reads that were aligned to regions within 5,000 bps of the off-predicted target sites were quantified. For LAM-HTGTS analysis, reads were checked for correct priming and then aligned to the AAV ITR sequence to quantify AAV integrations. The unaligned reads were end-to-end mapped to in silico–generated outcomes. Remaining unmapped reads were then trimmed to the Cas9 target sites and globally aligned to human genome (hg38). This analysis allows for quantification of AAV integrations, wild type (non-targeted), indel and deletion, inversion, HDR, and translocation events.

## Recipes

1. **P3 electroporation buffer**
  Freshly prepare 20 μL of P3 Primary Cell electroporation buffer by adding 3.6 μL of supplement to 16.4 μL of P3 Nucleofector^TM^ solution (Lonza).
  Mix the buffer by vortexing and briefly spin down at 280 × *g* for 1 min.
  Store at room temperature until use.
2. **Serum-free StemSpan^TM^ SFEM II medium**
  Serum-free StemSpan^TM^ SFEM II medium (Stemcell) supplemented with human SCF (100 ng/mL), human TPO (100 ng/mL), human FLT3L (100 ng/mL), human IL-6 (100 ng/mL), 35 nM UM171, and 0.75 mM SR1.
  Store at 4 °C for one week.
3. **70% ethanol**
  Freshly mix 30% (v/v) with 70% (v/v) ethanol.
4. **50% (wt/vol) PEG 8000**
  Dissolve 1 g of PEG 8000 in 2 mL of H_2_O and mix the solution with a thermomixer at 56 °C.
  Filter the solution through a 0.22 μm filter, prepare aliquots, and store them at -80 °C.
5. **5 M NaCl**
  Dissolve 292.2 g of NaCl in 800 mL of H_2_O and adjust the total volume to 1 L.
  Autoclave and store the solution at room temperature.
6. **2.5 N NaOH**
  Dissolve 10 g of NaOH in 100 mL of H_2_O.
7. **0.5 M EDTA**
  Dissolve 186.1 g of EDTA·2H_2_O in 800 mL of H_2_O.
  Stir vigorously on a magnetic stirrer.
  Adjust the pH to 8.0 using 2.5 N NaOH and fill up to 1 L.
  Autoclave and store the solution at room temperature.
8. **1 M Tris-HCl (pH 7.4)**
  Dissolve 121.14 g of Tris in 800 mL of H_2_O.
  Stir vigorously on a magnetic stirrer.
  Adjust the pH to 7.4 using HCl and fill up H_2_O to 1 L.
  Autoclave and store the solution at room temperature.
9. **1 M Tris-HCl (pH 8.0)**
  Dissolve 121.14 g of Tris base in 800 mL of H_2_O.
  Stir vigorously on a magnetic stirrer.
  Adjust the pH to 8.0 using HCl and fill up H_2_O to 1 L.
  Autoclave and store the solution at room temperature.
10. **1× TE buffer (pH 8.0)**
  10 mM Tris-HCl (pH 8.0)
  1 mM EDTA (pH 8.0)
  Store the solution at room temperature
11. **B&W buffer**
  1 M NaCl
  5 mM Tris-HCl (pH 7.4)
  1 mM EDTA (pH 8.0)
  Filter and store the solution at room temperature
12. **20 mM hexaamminecobalt(III) chloride (HexCo)**
  Dissolve 5.3 g of HexCo in 1 L of H_2_O to prepare a 20 mM working solution. Store the solution at room temperature.
13. **50 mM bridge adapter**
  Anneal two oligonucleotides [sequences are identical to Hu et al. (2016)] by heating the 400 mM (total) mixture in 25 mM NaCl, 10 mM Tris-HCl (pH 7.4), and 0.5 mM EDTA at 98 °C and ramp down at a rate of 1 °C/min to room temperature using the thermocycler. Dilute the mixture to 50 mM in nuclease-free water, aliquot, and store at -20 °C.
